# Sensors Fusion and Multidimensional Point Cloud Analysis for Electrical Power System Inspection

**DOI:** 10.3390/s20144042

**Published:** 2020-07-21

**Authors:** Vinicius F. Vidal, Leonardo M. Honório, Felipe M. Dias, Milena F. Pinto, Alexandre L. Carvalho, Andre L. M. Marcato

**Affiliations:** 1 Electrical Engineering Department, Federal University of Juiz de Fora, Juiz de Fora 36036, Brazil; vinicius.vidal@engenharia.ufjf.br (V.F.V.); felipe.dias@engenharia.ufjf.br (F.M.D.); andre.marcato@ufjf.edu.br (A.L.M.M.); 2 Electronics Department, Federal Center for Technological Education of Rio de Janeiro, Rio de Janeiro 20271, Brazil; milena.faria@engenharia.ufjf.br; 3 MRS Logistica, Juiz de Fora 36060, Brazil; alexandre.carvalho@mrs.com.br

**Keywords:** thermal inspection, multidimensional point cloud, sensor fusion, autonomous inspection, infrared noise filtering

## Abstract

Thermal inspection is a powerful tool that enables the diagnosis of several components at its early stages. One critical aspect that influences thermal inspection outputs is the infrared reflection from external sources. This situation may change the readings, demanding that an expert correctly define the camera position, which is a time consuming and expensive operation. To mitigate this problem, this work proposes an autonomous system capable of identifying infrared reflections by filtering and fusing data obtained from both stereo and thermal cameras. The process starts by acquiring readings from multiples Observation Points (OPs) where, at each OP, the system processes the 3D point cloud and thermal image by fusing them together. The result is a dense point cloud where each point has its spatial position and temperature. Considering that each point’s information is acquired from multiple poses, it is possible to generate a temperature profile of each spatial point and filter undesirable readings caused by interference and other phenomena. To deploy and test this approach, a Directional Robotic System (DRS) is mounted over a traditional human-operated service vehicle. In that way, the DRS autonomously tracks and inspects any desirable equipment as the service vehicle passes them by. To demonstrate the results, this work presents the algorithm workflow, a proof of concept, and a real application result, showing improved performance in real-life conditions.

## 1. Introduction

Electrical companies rely on a vast infrastructure to safely operate their business, requiring periodic inspection and maintenance. This scenario is especially critical for the electrical power transmission and distribution network, which can reach thousands of kilometers. There are several different types of inspections. However, some electrical equipment faults create thermal stress points due to a resistance increase, which can be detected through thermal data analysis before a major contingency occurs. Usually, these inspections are performed manually by trained personnel with several disadvantages,such as high cost, long time demand, risks to human life, and human failure. The application of automated inspection methods can reduce the problems as mentioned above.

Many different methods can be applied to perform automated condition inspection [1]. For instance, in Reference [2], a few different techniques are analyzed, such as the application of vibration sensors and torque monitoring. Despite good results, those techniques require the implementation of dedicated sensors, and the deployment of sensors network to allow data storage and analysis, increasing cost and complexity.

Moreover, computer vision techniques present good alternatives for many different scenarios. In the last years, various studies were proposed for defects detection in equipment through computer vision. In Reference [3], an Unmanned Aerial Vehicle (UAV) equipped with two cameras performs a three-dimensional reconstruction of an industrial facility for future inspection. Another application of image processing is found in Reference [4]. The authors proposed an image processing system for defects detection in paved streets based on color and texture information. Reference [5] places the camera on top of a running train, and an image processing system checks the distance between the rails enabling the faults detection.

A common characteristic in the previous works is the fact that they rely purely on the visible spectrum, which is a disadvantage once most failures on electrical parts generate heat from impedance increase or excessive load. Moreover, thermal imaging has been used in the last years to prevent possible faults in electrical units. According to Reference [6], bad connections, unbalanced loading, excessive use, or wear out are some of the factors that cause thermal stress in electrical components, which can be detected by hotspots in thermal images. Thermal cameras and 3D thermal models have already been used, separately, in a wide range of applications, such as in the sectors of building inspection [7,8], defect detection [9], and energy efficiency analysis [10,11]. However, just a few works have already applied the combination of 3D modeling with thermal data, for example, Reference [12], which still are limited and not used in an online fashion. Other works have already applied thermal inspection to electrical equipment, such as Reference [13]. However, there is no combination of 3D reconstruction, limiting its potential.

The main contribution of this research is the design and implementation of a Multidimensional Point Cloud Analysis (MPCA) methodology. The system is composed of an autonomous Directional Robotic System (DRS) robot embedding two RGB and one thermal camera. It has the capacity to move in the tilt and pan directions. The application of a Simultaneous Localization and Mapping (SLAM) methodology allows it to evaluate the robot’s exact position to point the cameras, generating n-dimensional models of previously selected objects. By calibrating the cameras, it is also possible to project the temperature readings from the thermal image into the 3D model. This proposal generates a Multidimensional Point Cloud (MPC) through the association of each spatial point to a thermal value. Moreover, each 3D point will have a temperature profile regarding its different position readings by repeating this process from different poses. The analysis of these profiles can indicate misreading due to infrared reflection, improving the quality of the measures. Finally, once in thermal inspection, the problem localization is a reliable indication of the diagnosis itself, this approach facilitates the correct analysis and helps the diagnosis.

To demonstrate the effectiveness of this approach, the MPC will be built over a vehicle for inspection of an electrical power distribution system. These research contributions can be summarized as follows:

- A mechanism to provide reliable 3D/thermal (MPC) information for equipment inspection.
- A real application of automated electrical power distribution line inspection using a DRS along with stereo and thermal cameras to result in real-time MPCs.
- An optimized approach to process multiple MPC to filter thermal misreadings.

The remainder of this research is organized as follows—Section 2 presents a brief review of the related work highlighting the state-of-the-art in SLAM and thermal inspection. Section 3 details the architecture and its foundations for 3D reconstruction, Section 4 shows the system assembly and deployment, Section 5 shows the proposed experiments with a proper discussion of the results. The concluding remarks and future work are conducted in Section 6.

## 2. Background and Related Works

Inspections of electrical power transmission/distribution related equipment are performed based on four main methods, that is, helicopters, UAVs, road vehicles, and manually by operators. The use of helicopters and UAVs has the disadvantage of being relatively far from the subjected inspection area. The applications of helicopters can also be quite expensive, while some UAVs present low battery time that may limit its use. Manual inspection by operators is too costly and can be time-consuming, making it impractical in some situations. Thus, the use of road vehicles with mounted equipment becomes a suitable option for this type of inspection, especially at the distribution level.

Inspection based on vehicle-mounted systems is used in many areas. The most common use of this technique is concentrated in the rail inspection on works such as Reference [14]. However, some studies have applied these techniques to inspect tunnels [15], platforms, and ancillary equipment [16].

Many challenges arise in the application of computer vision to perform autonomous inspections. Those challenges are related to some practical aspects of the robot. For example, when determining the robot position accurately, performing proper 3D reconstruction using multiple data from the visual and thermal camera hardware, or controlling the primary function of the robotic system. The following subsections discuss related works using the techniques proposed in this research.

### 2.1. Precise Localization Using Visual Odometry (VO)

The current proposal of this work depends on the robot’s real-time precise position. This information is used to aim the system to a desirable and previously know object, and to fuse each point cloud into the final n-dimensional model.

According to Reference [17], environment mapping, such as the SLAM technique, is a fundamental approach to guarantee safe path planning. In the last decades, a few well-known SLAM methods were developed. The work of Reference [18] introduced a technique called LSD-SLAM. This method performs SLAM from direct image alignment using a monocular camera. The result is a pose graph without the scale drift problem inherent in monocular vision. The authors of Reference [19] proposed an algorithm that outperformed LSD-SLAM in location and mapping with 3D semi-dense reconstruction and VO. This process uses information from stereo vision and other sensors, such as IMU, fused, and filtered. They presented qualitative and quantitative results in real datasets, running in real-time on a CPU. Another SLAM algorithm is found in Reference [20] introduces the ORB-SLAM2 open-source algorithm, presenting a complete solution for SLAM, that is, using monocular, stereo, or RGB-D cameras. The results overcame LSD-SLAM methods in metrics such as time and rotation error in many KITTI benchmark datasets, with the advantage of accuracy and efficiency and also running on CPU. Note that some modern techniques for VO calculated with high frame rate cameras guarantee good quality mapping and location from relatively slow movements, requiring less processing capacity from the hardware [17]. Applications assessing its effectiveness can be found in the most variety of vehicle types and environments, as seen in References [21,22].

Stereo Vision is a consolidated technique for robotics applications, computing both 3D information maps and VO for the robot in the environment, even in real-time. Some works using and assessing stereo vision SLAM results can be found in References [23,24], where it is compared to other sensors and used for obstacle avoidance as well. The work of Reference [25] uses stereo vision to perform SLAM in multi-robot team control. The literature has also highlighted the effectiveness of this technique in outdoor scenarios.

Most state-of-the-art methodologies use SLAM for autonomous navigation or complex environments reconstruction, so this works needs a robust, still lightweight SLAM approach methodology for fast and accurate spacial results. For this reason, a modified ORB-SLAM2 VO  [20] running in a closed loop with a traditional Kalman filter is applied for the robot localization.

### 2.2. Thermal Inspection in Engineering

There are several works in the literature regarding the use of infrared cameras for industrial and maintenance applications. One noticeable field is the inspection in buildings and constructions pursuing heat leakage or electrical equipment issues. The research presented by Reference [26] brings a solution for generating thermal building information models by fusing information of an infrared camera with a 3D laser scanner. The equipment returns a model containing the temperature distribution in the interior of each room for further analysis.

Regarding infrared thermography for electrical equipment, some studies have presented solutions and results from the image data itself. The work developed by Reference [27] shows quantitative and qualitative methods for analyzing defects from thermal images and gathered temperature values, as much as considering their automatic recognition. In Reference [28], a fuzzy system is applied automatically to recognize and classify equipment failures from thermal image inputs. Induction motors are the focus in Reference [29], where the authors developed an algorithm to classify the faults observed in the thermal images.

The use of thermal and visible inspection is vastly applied in the railroad industry, where both the rail and vehicle conditions, as much as the surrounding distribution lines, are subjected to fault risks that can be prevented by analyzing infrared images. Reference [30] mentioned that the inspection labor is done many times by land in a non-effective manner, and brings a solution using a UAV for image data gathering in an automated fashion. Besides the applicable approach, it still relies on many conditions, for example, weather, vehicle line of sight, and channel links quality, not to mention pilot trained personnel.

Using RGB and thermal cameras, Reference [31] proposed a solution for correct thermal image registration with a novel image descriptor combining visual and thermal information to inspect the components. The results are used for thermal issues detection, while still in the 2D aspect world. Fusing the data acquired by thermal and RGB-D cameras, Reference [32] presented a device that scans real objects in 3D and returns the registered point cloud with thermal information as an ultimate result. All the process is described, from camera parameters calibration and motion estimation to data fusion into the point cloud. Still, the process is performed manually and not suitable for many external applications. In Reference [33], the authors presented a system to generate 3D thermal models with a combination of a stereo, an RGB, and a thermal camera. Besides three cameras, the stereo one is not used for the 3D models, but to generate the odometry data. Thus, this system has the same limitations as Reference [32].

Therefore, the motivation of this work is to use the benefits of thermal analysis in distribution line components in an automated fashion. This motivation, combined with a lightweight algorithm that calculates point clouds and VO to perform SLAM, provides an n-dimensional thermal and visual model of a given component, with data acquired from different poses.

## 3. The MPCA Approach

**Figure 1:**
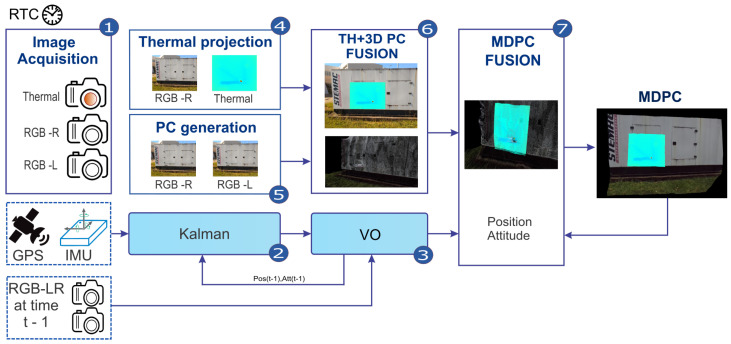
Methodology Diagram.

As stated before, the proposed approach fuses temperature data from a thermal camera with a 3D point cloud generated by a stereo camera for further analysis. Figure 1 presents a global overview of the proposed methodology divided into its seven processes. The system performs concurrent processing with delivered responses varying from hard real-time to offline. The most critical part is the data acquisition and, therefore, has priority over all the others. A real-time trigger controls the synchronization of both visual and thermal images, along with GPS and IMU data. The cameras are connected to the main computer through an Ethernet cable and have global shutter capability. All the processes are listed in Table 1, showing their respective priority, time requirements, and description.

### 3.1. Synchronization Process and Cameras Calibration

Literature present a vast amount of calibration techniques. Considering RGB cameras, the calibration process usually includes a checkerboard pattern with a known square size due to its simplicity. However, the same approach cannot be replicated for thermal cameras because the images of a standard checkerboard pattern do not have contrast, that is, temperature variation, for calibration. Therefore, the literature shows several methods for thermal camera calibration. In Reference [34], a halogen lamp heats a standard checkerboard to obtain thermal contrast. In Reference [35], a 9 × 9 small bulb matrix is used as the calibration pattern. This matrix generates a set of 100 reference points easily mapped from one image to another.

Different from what is presented in the literature, this work has chosen a different calibration system approach. A checkerboard pattern was printed in a plastic paper and attached to a squared piece of glass. An halogen bulb lamp heats the back of a personalized pattern, and then, the calibration process is performed.

Moreover, the calibration process defines each camera definition, its intrinsic K matrix, the radial distortion and its relative position.

For the rest of the work, it is considered that the images are corrected by the radial distortion. Finally, the cameras are synchronized through a master-slave system. A real-time clock sends a 10 Hz signal, triggering the cameras. A watchdog layer ensures that the three cameras are always synchronized by checking their time-stamps and choosing to publish or discard the images.

### 3.2. Visual Odometry Algorithm

The open-source ORB-SLAM2 algorithm was chosen in this research to calculate the VO. As seen in the work of Reference [20], this algorithm is composed of three main threads. The first one is responsible for calculating feature-based camera odometry in every frame. Besides, it minimizes the back-projection error using motion-only Bundle Adjustment (BA). The second thread computes and optimizes the local map with the use of local BA. Finally, the last one deals with loop closures employing a pose-graph optimization.

The robot is submitted to discrete trajectories of a few meters length for data acquisition throughout the current path since this research intends to monitor electrical equipment along the distribution line. Therefore, it is not expected for the path to repeat itself. Thus only the Localization Mode of ORB-SLAM2 algorithm is applied. In this mode, both second and third threads are deactivated for performance. Moreover, odometry relies on matches between the current frame’s ORB features and the 3D points calculated from stereo depth in the past frames to evaluate the motion. This algorithm separates the matched points in two categories to achieve better results in odometry, that is, close and far depth points. The points are separated by a threshold of *X* times the baseline distance. This method guarantees that close points are triangulated for more accurate translation estimation, while still using far points for rotation when seen in multiple views. For our application, a value of 100 was empirically defined in the inspection track.

As this process alone can integrate error along the path, it was proposed to use the VO algorithm in a closed loop with an Extended Kalman filter with colored electromagnetic interference, as shown in Reference [36]. For every pair of synchronized images, the VO is calculated in parallel to the stereo point cloud. It is vital for the later registration process algorithm and thermal 3D data acquisition.

### 3.3. Thermal Projection

After the calibration process, Equation (1) maps the thermal image $I^{th}$ to the visual RGB-R one $I^{v}$ , by mapping every pixel $p_{k}^{th}={\left(x^{th},y^{th}\right)}_{k}$ to its corresponding location $p_{k}^{v}={\left(x^{v},y^{v}\right)}_{k}$ at every instant *k*. The values of $x^{v},y^{v},x^{th},y^{th}$ must be inside their respective cameras resolutions.

Finally, the mapping process uses the homogeneous transformation matrix $H_{v}^{th}$, which comprises the rotation **R**, translation **t** and distortion elements **d** between both image sources [37]. Finally the scalar *s* that deals with the final thermal image resolution, as a function $f:p_{k}^{th}\mapsto p_{k}^{v}$, described in Equation (1). Formal definition of all variables are shown in Appendix A.

(1) $$\left[\begin{array}{c} x^{\prime v}\\ y^{\prime v}\\ w^{v} \end{array}\right]=sH_{v}^{th}\left[\begin{array}{c} x^{th}\\ y^{th}\\ 1, \end{array}\right]$$

where

(2) $$H_{v}^{th}=\left[\begin{array}{cc} R & \frac{t}{s}\\ \frac{d}{s} & \frac{1}{s}. \end{array}\right]$$

The result is defined up to a scale related to $w^{v}$, so the value of $p_{k}^{v}$is calculated in Equation (3).

(3) $$p_{k}^{v}=\left[\begin{array}{c} x^{\prime v}/w^{v}\\ y^{\prime v}/w^{v} \end{array}\right].$$

The evaluation of $H_{v}^{th}$ is given by the optimization problem shown in Equation (4), where *N* is the number of reference points extracted from each of the nP pictures taken from the board at the individual camera calibration process, $I^{vr}$ is the image of the right picture, $I_{a}\left(p_{b}\right)$ represents the point $p_{b}$ from image $I_{a}$

(4) $$\begin{array}{cc} Min & f\left(s,R,t,d\right)=\sum_{i=1}^{nP}\sum_{j=1}^{N}\left|\left|I_{i}^{vr}\left(p_{j}^{vr}\right)-sH_{v}^{th}I_{i}^{th}\left(p_{j}^{th}\right)\right|\right|, \end{array}$$

where Equation (4) is optimized by using the Levenberg-Marquardt algorithm.

**Figure 2:**
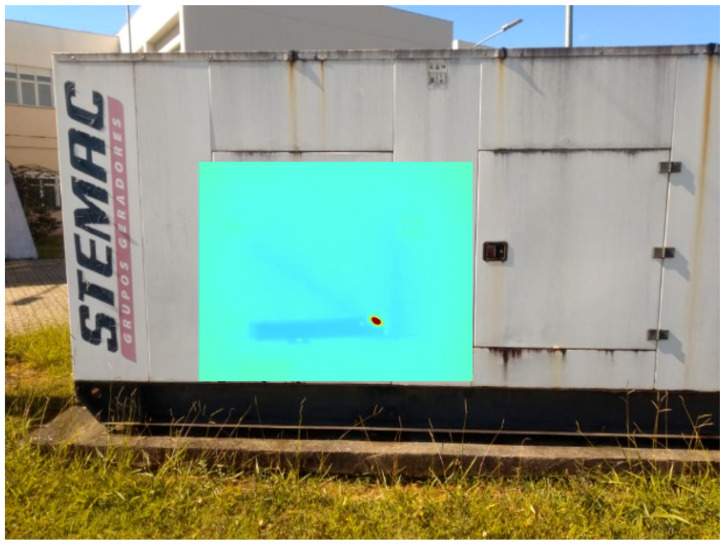
Thermal and Visual homography transformation result.

It is important to note that the Field of View (FoV) and resolution of the thermal camera are both lower than the visual’s ones. The resolution is a cost/benefit choice, while the FoV was designed in this way to keep a good thermal resolution for distant objects. The result is a window of thermal projection inside the visual information, which will be assigned as $W^{th}$ and has the same properties of $I^{th}$. A final observation is that the utilization of the entire RGB image facilitates VO and point cloud registration and fusing. Figure 2 shows a result of the thermal projection process.

### 3.4. Point Cloud Generation

The stereo algorithm used to compute the point cloud is based on Reference [38]. From a pair of images, the following steps are performed. First, the images are rectified by using the parameters obtained from the calibration process. Then, to reduce saturation problems between two different points of view, the images are converted to grayscale and then normalized to enhance texture and diminish possible differences in illumination. A sliding window $W\in Z^{7x7}$ calculates the new color value of color for its central pixel $w_{c}^{\prime }\in Z$ as in Equation (5).

(5) $$w_{c}^{\prime }=\min\left[\max\left(w_{c}-\bar{w}\right),w_{cap}\right],$$

where $w_{c}\in Z$ is the original color value for pixel *i*, $\bar{w}\in R$ is the average color values of pixels in the slide window *W*, and $w_{lim}\in Z$ is a predetermined limit to avoid negative values.

The next step consists in comparing the similar points from left to right image using the Sum of Absolute Difference (SAD) operation from a fixed window $W^{L}$ in the RGB-L image to a sliding $W^{R}$ in the RGB-R one (both converted to grayscale), for a range of pixels previously defined as the disparity range dr in the *x* direction (Equation (6)). After performing the operation, the lowest value $M_{min}$ is considered as a match candidate between $p_{L}^{v}$ and $p_{R}^{v}$ pixels from RGB-L and RGB-R images, respectively. If $M_{min}$ satisfies the uniqueness ratio un threshold in Equation (7) for all the other $M_{i}:i\in dr$, the match is considered as valid, and the pixel disparity *d* between $p_{L}^{v}$ and $p_{R}^{v}$ (Equation (8)) is annotated in the disparity map τ as the difference between the pixels *x* coordinates.

(6) $$M_{i}=\sum_{x=1}^{m}\sum_{y=1}^{n}\left|w_{x,y}^{R}-w_{x,y}^{L}\right|,i\in dr,$$

(7) $$un>\frac{M_{i}-M_{min}}{M_{min}},$$

(8) $$d=x_{R}-x_{L}.$$

Finally, the depth is calculated via triangulation operation for every $p_{i}^{\tau }$ pixel value in the disparity map, as described in Equation (9).

(9) $$Z_{i}=\frac{fb}{d_{i}},\forall p_{i}\in \tau ,$$

where $Z_{i}$ is the depth for the pixel’s corresponding point in 3D $P_{i}^{v}\in R^{3x1}$, *f* is the RGB cameras focal length and *b* is the stereo rig baseline. The instantaneous point cloud $C_{k}^{v}$ for instant *k* is composed of the group of $P_{i}^{v}$ originated from τ, and is calculated for every $p_{i}^{\tau }\in \tau$ corresponding $x_{L}^{v}$, $y_{L}^{v}$ and $d_{i}$ in Equation (10).

(10) $$P_{i}^{v}=\left[\begin{array}{c} {x_{R}^{v}}_{i}-c_{x}\\ {y_{R}^{v}}_{i}-c_{y}\\ f, \end{array}\right]\frac{b}{d_{i}}$$

where $d_{n}$ is the disparity for each match, and $c_{x}$ and $c_{y}$ are the principal point coordinates in the RGB-R image.

**Figure 3:**
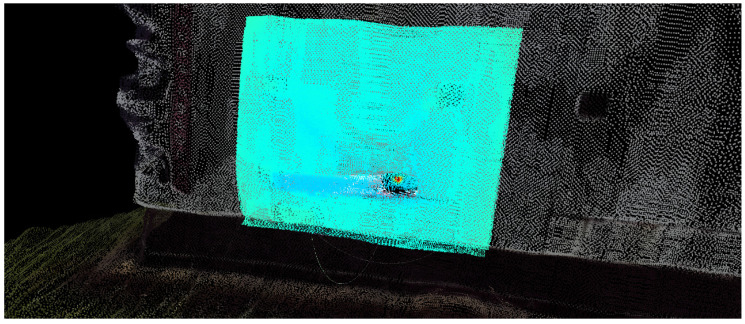
Instant thermal reconstruction.

In possession of the intrinsic matrix $K^{v}\in R^{3x3}$ for the RGB-R image containing the focus and principal point values *f*, $c_{x}$ and $c_{y}$, respectively, the points $P_{i}^{v}$ from $C_{k}^{v}$ can be projected into the image plane to its respective pixel location $p^{\prime v}$ in homogeneous coordinates as in Equation (11). Again, to get final coordinates, one must divide the result by $w^{v}$ and get $p^{v}$, following Equation (3). Figure 3 shows the final instant thermal 3D reconstruction.

(11) $$\left[\begin{array}{c} x^{\prime v}\\ y^{\prime v}\\ w^{v} \end{array}\right]=K^{v}\left[\begin{array}{c} X^{v}\\ Y^{v}\\ Z^{v}, \end{array}\right]$$

where

(12) $$K^{v}=\left[\begin{array}{ccc} f & 0 & cx\\ 0 & f & cy\\ 0 & 0 & 1. \end{array}\right]$$

### 3.5. Accumulated N-Dimentional Point Cloud

The point clouds $C^{v}$ and $C^{th}$ must be registered correctly regarding the world inertial frame. This is performed through a homogeneous transformation matrix given by the VO algorithm. Consider ${}_{v}T_{k}^{in}$ as the odometry transformation from the origin of the inertial frame to the RGB-R camera frame. In a first moment, the registration of $C_{k}^{v}$ (with *N* points $P_{i}^{v}$) concept could be done by stacking the clouds after the homogeneous transformation for every instant *k*, building the visual accumulated point cloud ${Ac}^{v}$ as in Equation (13) for a total of *K* instants.

(13) $$Ac^{v}=\sum_{k=1}^{K}\sum_{i=1}^{N}\left({}_{v}T_{k}^{in}\left[\begin{array}{c} X_{i}^{k}\\ Y_{i}^{k}\\ Z_{i}^{k}\\ 1, \end{array}\right]\right)$$

where

(14) $${}_{v}T_{k}^{in}=\left[\begin{array}{cccc} r_{1} & r_{2} & r_{3} & t_{x}\\ r_{4} & r_{5} & r_{6} & t_{y}\\ r_{7} & r_{8} & r_{9} & t_{z}\\ 0 & 0 & 0 & 1. \end{array}\right]$$

**Figure 4:**
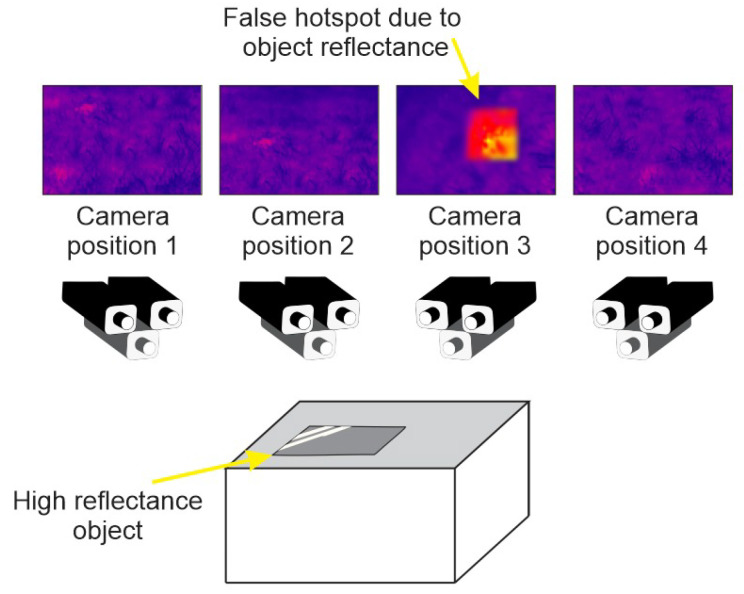
Algorithm to recognize false measurements removal.

Analogously, there should exist an accumulated thermal point cloud $Ac^{th}$ formed by the addition of every $C_{k}^{th}$ cloud. Therefore, each 3D visual point with a thermal projection is associated with an n-dimensional temperature array, where n is the number of times that each 3D point is found in a pose. It is interesting to mention that, due to occlusions or other factors, the size of n changes from point to point. It means that points that are captured more times have a larger temperature vector. There are two possible approaches to deal with and analyze these accumulated thermal readings. One is to generate the n-dimensional vectors, use sophisticated analysis to find a diagnosis or operate a filter at each new entry, and store just one filtered value. As it is not the proposal of this work to analyze with filter is the best, the second approach is adopted. The final registration process uses a min filter to remove false temperature measurements illustrated by Figure 4. In an instant *k*, the new point cloud $C_{k}^{th}$ is submitted to a *KD-Tree* search process for corresponding points in $Ac^{th}$. If neighbors are within a radius, the point temperatures from different instants are compared, and the lowest one is chosen. In case no neighbor is found, this new point is added to $Ac^{th}$. The process is described in Algorithm 1.
**Algorithm 1** Temperature correction algorithm.$C^{th}$ = new_point_cloud() $C^{transf}=transform\left(C^{th},{}^{v}T_{in}\right)$

**for**
$P^{th}$ ∈ $C^{transf}$
**do**    neighbors = Kd_tree_search($P^{th}$, $Ac^{th}$, thresh_radius)    **if** (neighbors>0) **then**        temperatures = get_temperatures(neighbors, $P^{th}$)        temp = lowest_temperature(temperatures)        $P^{th}.temperature$ = temp    **else**        $Ac^{th}$ += $P^{th}$    **end if** **end for**


**Figure 5:**
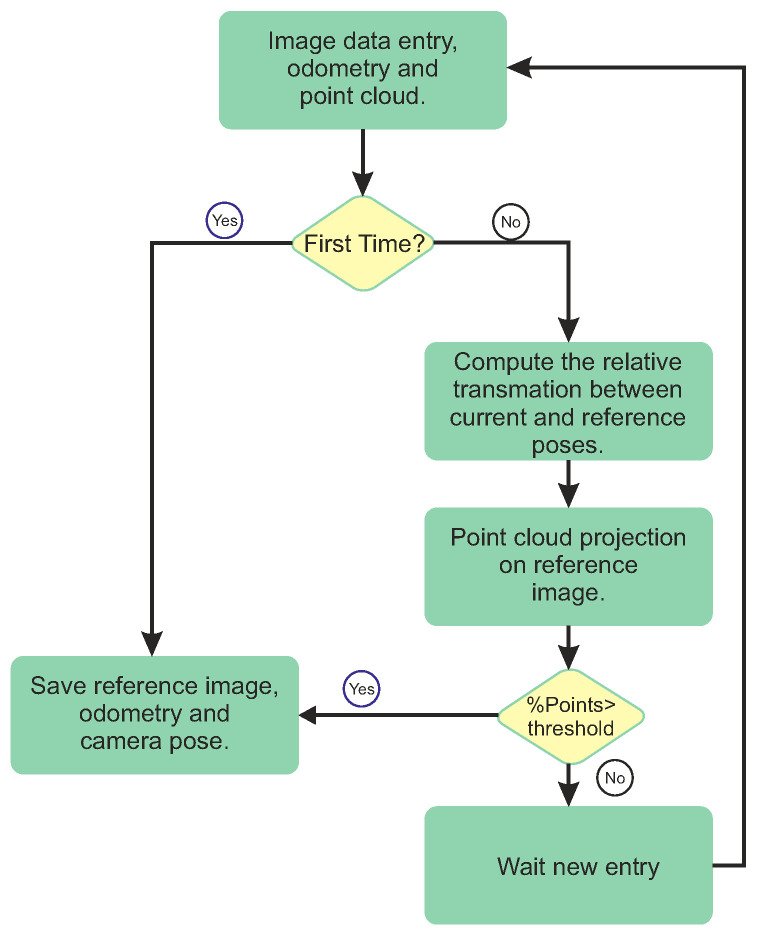
Cloud overlap analysis process.

Note that accumulating duplicated 3D points wastes computer memory and processing capacity without any improvement. Thus, this work uses an overlap calculation to avoid this problem. This process uses the RGB-R camera pose to evaluate the homogeneous transformation ${}_{v}T_{ref}^{in}$. The relative movement and its respective odometry ${}_{v}T_{k}^{in}$ are computed for the new point cloud $C_{k}^{v}$, which is projected in the reference pose by using Equation (15). If this point cloud meets the thresholds of a minimum number of new points and a minimum distance from the accumulated pose, the algorithm considers $C_{k}^{v}$ as a good point cloud. In such a case, it starts the registration process considering the thermal pair $C^{th}$. The odometry measurement ${}_{v}T_{k}^{in}$ is taken as a new odometry reference ${}_{v}T_{ref}^{in}$, and the process restarts. The final accumulated result is obtained by applying Equation (14) to this newly accepted point cloud. Figure 5 presents a flow chart of this process, where at the first time that a set of images is acquired, it is considered as reference frame until a threshold is met and a new reference frame is considered.

(15) $$p^{\prime v}=K^{v}{\left({}_{v}T_{ref}^{in}\right)}^{-1}{}_{v}T_{k}^{in}\left[\begin{array}{c} X_{i}^{v}\\ Y_{i}^{v}\\ Z_{i}^{v}\\ 1. \end{array}\right]$$

**Figure 6:**
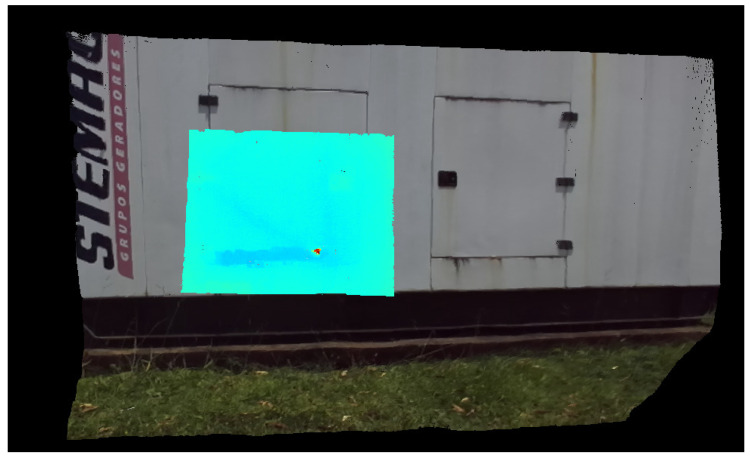
Instant N-Dimensional reconstruction.

An example of accumulated N-Dimensional point cloud with focus on the reflected misreading temperature, before correction, can be seen in Figure 6.

## 4. Visual System and Robot Description

**Figure 7:**
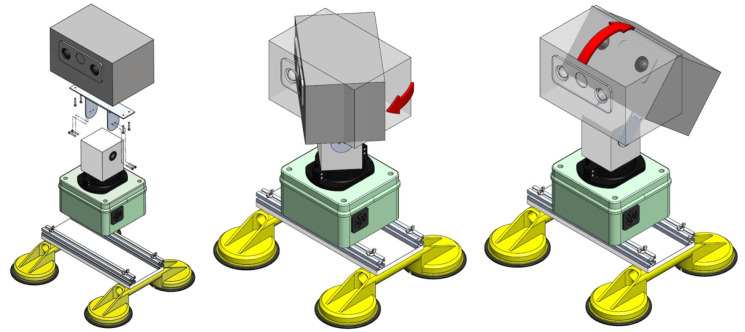
Robot’s Schematic view, with Pan and Tilt capabilities illustration.

**Figure 8:**
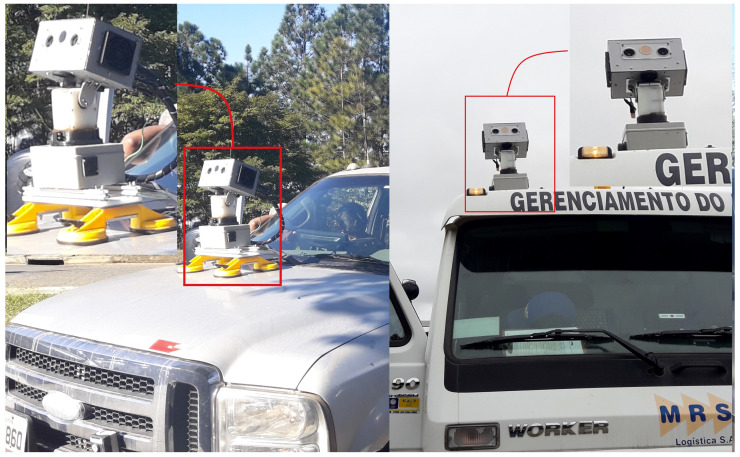
Robot assemble in two different configurations and inspection vehicles.

Figure 7 presents the robot developed for this application, namely Wally3. It is composed of two main structure parts, that is, body and head, which guarantee tilt and pan capabilities, as seen in Figure 8. The robot is mounted on top of a vehicle capable of driving along the railway to automatically monitor the distribution lines on the sideways. In the end, it is composed of vision, automatic orientation control, power distribution, and processing core systems.

**Figure 9:**
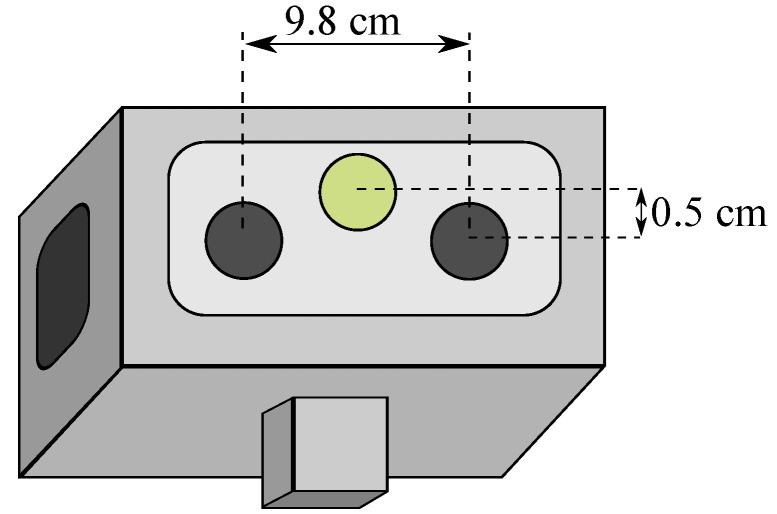
Scheme for Stereo and Thermal Cameras Positioning.

The vision system is coupled on Wally3’s head, with a stereo pair of Allied Vision’s MAKO cameras on both sides and a FLIR A65 thermal camera in the center of the scheme. The calibration process calculates the cameras intrinsic and the extrinsic parameters relating each camera to the other two. The visual cameras capture images up to 20 Hz rates, with 1600 × 1200 resolution. Regarding the thermal camera, images capture up to 13 Hz rates, with 640 × 512 resolution. Figure 9 brings the relative position and spacing between the cameras in the robot. All cameras have global shutter or similar capture systems and are synchronized by a real time clock, meaning that images are only acquired when all cameras are ready. This approach mitigate problems such as shutter deformations and the parallax motion effect, which means that, objects closer to a moving camera tend to blur. Moreover, if the parallax field of view effect is considered, it is essential to remember that only the visual cameras are used to generate point clouds, and the thermal one is used to project the temperature readings over the right-placed camera. Thus, the distance value of ≅ 10 cm provides a good trade-off between accuracy and compactness and having the thermal camera closer to the visual camera mitigates occlusion and other undesirable effects.

**Figure 10:**
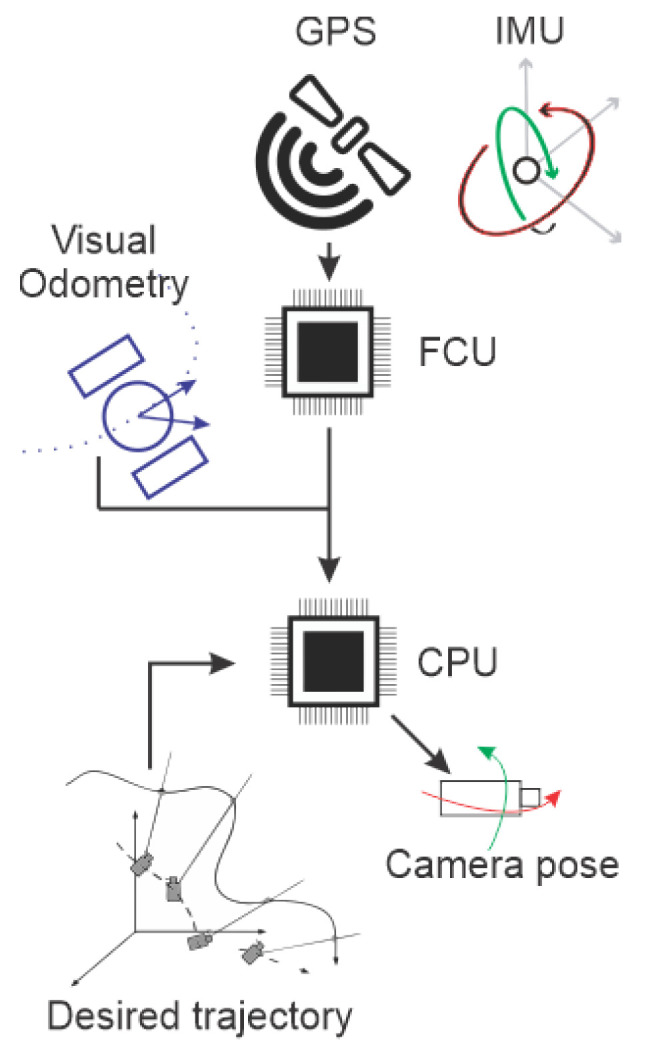
Camera pose control operation.

**Figure 11:**
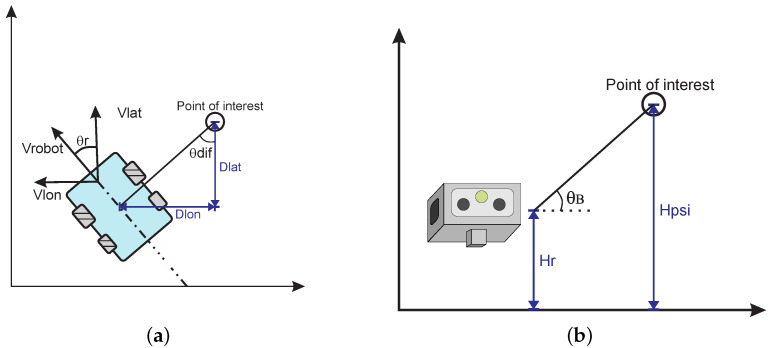
Automatic Orientation Scheme for the robot’s behavior when inside a Point of Interest predefined region. (**a**) Pan. (**b**) Tilt.

The automatic orientation control relies on a Pixhawk controller board placed inside the body and a GPS module. Figure 10 and Figure 11a,b illustrate the robot’s behavior for pan and tilt movements. First, the controller gathers data from inertial sensors and GPS to provide the robot’s position in the world, and so calculate its orientation relative to the point of interest. The relative angles are transmitted to the servos for pan and tilt adjustments, so the tracked point is always inside the robot’s Field of View (FOV). As the dynamics of the servos and their encoders are well known and reliable, both are used in the Kalman filter process to mitigate angular misreadings.

Due to many sources of electromagnetic interference emanating mainly from the vehicle’s communication system, once a certain number of satellites is observed by the GPS, the orientation in the world uses information provided by this sensor to fuse it with the compass readings [39] . The fusion process uses an Extended Kalman Filter with electromagnetic disturbs [36]. This is a viable approach once the vehicle only moves forward during the inspection. Equation (16) describes the new orientation sensor $\theta_{r}$ calculation in the world frame.

(16) $$\theta_{r}=atan2\left(v_{lon},v_{lat}\right),$$

where $v_{lon}$ and $v_{lat}$ stand for the velocity in the longitude and latitude directions, respectively.

Equation (17) is responsible for estimating the angle $\theta_{dif}$ from the vehicle to the point of interest in the world frame. Subsequently, Equation (18) gives the final smallest relative angle γ from vehicle’s forward-looking direction to the point of interest location.

(17) $$\theta_{dif}=atan2\left(d_{lon},d_{lat}\right)$$

(18) $$\gamma =warp180(\theta_{r}-\theta_{dif}),$$

where $d_{lon}$ and $d_{lat}$ are the difference in longitude and latitude coordinates from the point of interest to the robot. This new reading is incorporated into the CKF to evaluate the final orientation and position.

Finally, Equation (19) calculates the tilt angle β. It considers the distance from the vehicle to the point of interest $D_{r\_poi}$ and the difference in height from where the robot cameras are ($H_{r}$) to the one estimated in the mission for the equipment to be inspected, namely $H_{poi}$:

(19) $$\beta =atan2(H_{poi}-H_{r},D_{r\_poi}).$$

After these calculations, the angle values are converted to Pulse Width Modulation (PWM) signal and sent to the actuators. Wally3 has two dedicated Dynamixel servo motors, where the model MX-106 is for pan and MX-64 is for tilt movements. They are both controlled internally by a PID controller, which is tuned for smooth movement during inspection not to disturb the image acquisition. The commands are sent to them at a rate of 6 Hz.

## 5. Results and Discussion

The experimentation methodology consists of moving the robot from a determined starting position to different inspection points. During the missions, the robot is subjected to different conditions to verify the autonomous capability of inspecting various equipment in the surrounds. The entire processing is performed in a computer with an Intel i7 core processor, running Ubuntu 16.04. The whole process is managed by the Robot Operating System (ROS) framework, responsible for organizing the algorithms for vision and orientation.

This research uses the developed Wally3 robot for methodology validation. Besides, two practical experiments were carried out to evaluate the effectiveness of the proposed methodology—(1) A reflective surface thermal inspection, to test the concept of sun light effect mitigation; and (2) A piece of equipment is inspected in the rail distribution line.

An important observation is that, after an extensive bibliographical research as shown in the introduction, it was not found any similar approach, which makes impossible to compare our results with any other recent approach. Instead, the current methodology will be compared with the results performed by a field expert.

### 5.1. Reflective Surface Inspection

**Figure 12:**
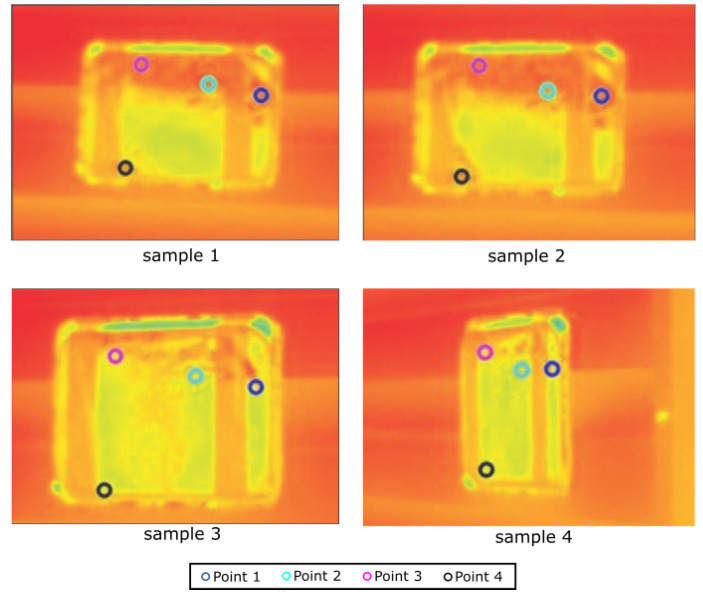
Image samples in different points of view, with 4 highlighted points.

**Figure 13:**
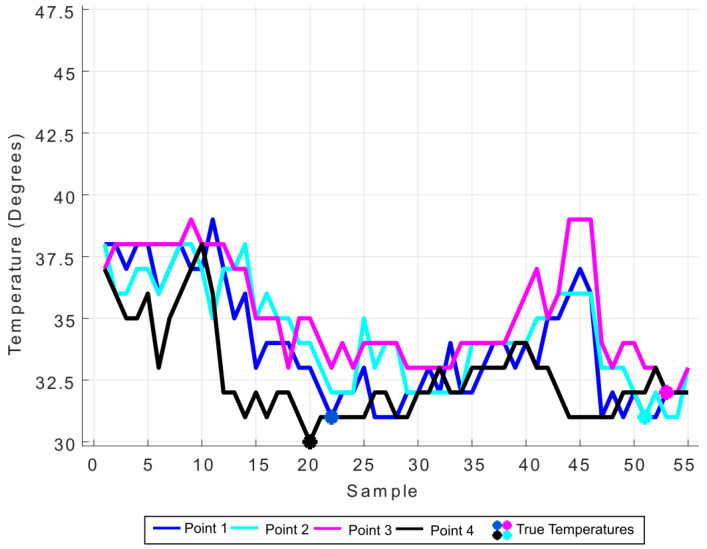
Temperature variation for each point along the test, with minimum temperature taken as real ones.

This setup allowed the heat diffusion through the plate and temperature monitoring. Note that the sunlight incidence on the board makes the temperature in determined spots increase depending on the point of view. To test the proposed approach, a random set of points was selected on the board surface for temperature analysis during a time interval. Figure 12 presents the points seen in four different sample instants, while a graph for their temperature variation is shown in Figure 13. Note a temperature difference of up to 30% from the lowest one gathered at some points, which could indicate a false hotspot and is avoided by the algorithm.

### 5.2. Real Application

Finally, the algorithm was tested in a real case scenario to inspect a 180 kVA autonomous diesel group generator was monitored during an active emergency operation for possible defects. It is a particularly crucial case once it is not typical for those types of equipment to enter in operation. Therefore when this situation happens, all related devices must be inspected as fast as possible. During the inspection, an infrared reflection was observed in a metallic piece attached to it. In a normal situation, this would demand a new service order for further analysis and correction.

**Figure 14:**
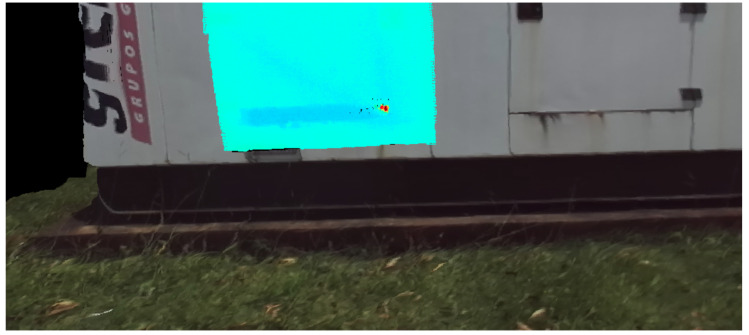
3D model with false positive reading due to sunlight reflection.

**Figure 15:**
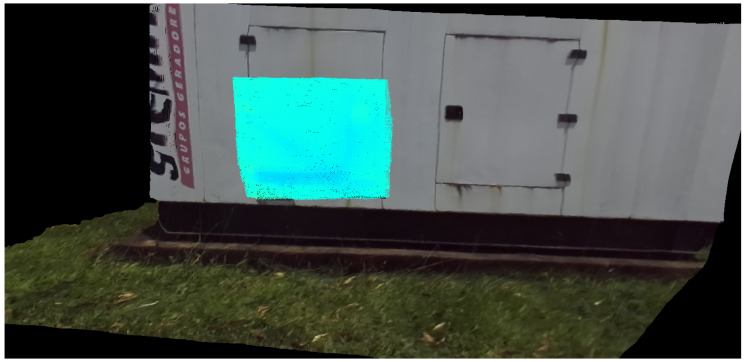
Temperature correction in the 3D model due to a reading from another angle.

Figure 14 shows the infrared interference as a red at the moment it is detected. Once it disappears, when the generator is seen from another angle in Figure 15, the plate returns to a uniform temperature color, which indicates that there was a false positive indication of defect in the past.

**Figure 16:**
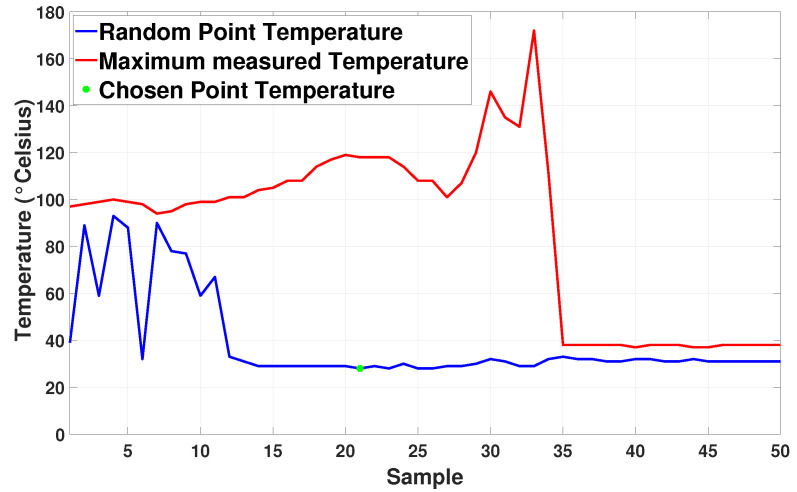
Temperature measurements for a random affected 3D point in blue and maximum reading for each image sample in red.

**Figure 17:**
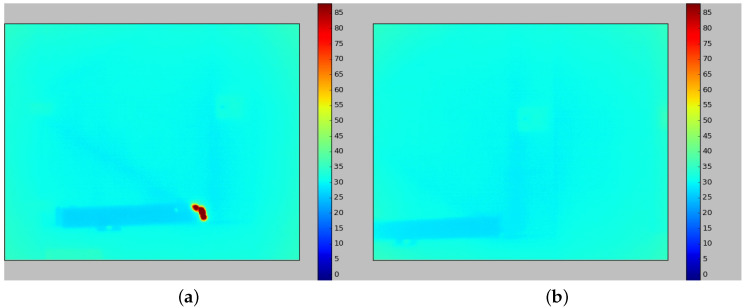
2D thermal pictures of the generator. (**a**) Hotspot in 2D thermal reading with temperature scale. (**b**) 2D thermal reading with no hotspot.

Figure 16 presents a graph of temperature evolution along the inspection. Two points are used to test the methodology in this real scenario. First, the blue line indicates the readings in a random 3D spot within a region of interest that presents infrared interference but is not the worst-case scenario. The second point represents the highest temperature variation found in the readings. This point is represented by the red line and it clearly shows the effect of external infrared interference over the readings. The filtered temperature is marked in green for the mentioned 3D point. Finally, Figure 17a,b presents 2D thermal pictures of the generator with and without sunlight reflection, respectively.

To compare the results, the same equipment was also analyzed by an expert. The qualitative result was exactly the same. However, Wally completed the inspection and diagnosis in less than 20 s while the expert took more than 10min. Considering, parking the car, deploying the equipment, process the readings, packing and leaving this process took 15 min.

Finally, an important observation, it that this approach only inspects parts of the object that is facing the road or equipments that any heat disturbance is propagated all over the object, such as insulators. Some cases in transmission lines, it is possible to circle around the object, but in distribution systems this is not always possible. But, even with this limitation, it is possible to reduce the overall inspection time in real routes in more than 30%.

### 5.3. Batch Inspection Mission

**Figure 18:**
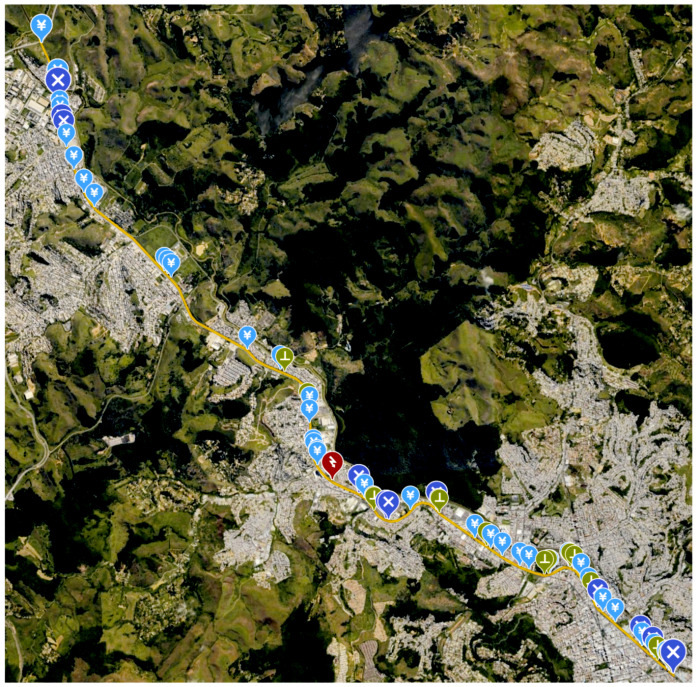
Batch Mission containing 50 POI with multiples elements with respective measured and filtered temperature.

**Figure 19:**
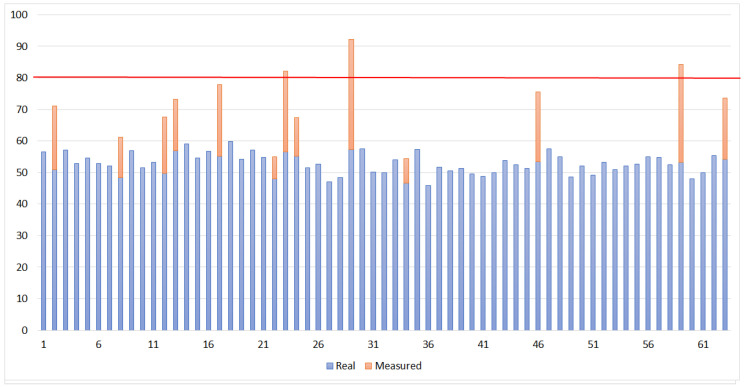
Measurement temperature (${}^{\circ }$C) profile and corrections.

As a multi-inspection performance evaluation, the system has executed a 16 km long real mission consisting of 62 points spread the railroad dedicated electrical distribution network. Each inspection point may present more than one equipment, and the goal is to keep the predictive maintenance updated by searching for potential faulty components . Normally, each equipment present a different operational temperature, however, as any fault related to thermal irradiation will result in a measure much higher than any normal operational point, a unique threshold of 8 ${}^{\circ }$C was set. Figure 18 shows the entire mission where the blue markers are transformers, purple are switches, green are Insulating shroud to underground systems and, finally, the red one is a small power substation. Figure 19 shows the measurement profile and temperature corrections. It is possible to see that was detected infrared interference in 13 cases, 3 of them indicating overheat. This situation would demand a ground team to check those locations for further analysis.

## 6. Conclusions and Future Work

This research has proposed an autonomous system composed of two RGB stereo cameras and one infrared camera to capture 3D visual and thermal models for each instant. These instantaneous models are integrated over time to create accumulated visual and thermal 3D models, which are used for inspection analysis. The solution adopted in this research is generic and has presented an effective alternative approach for autonomous inspections of any type of equipment and machinery in transmission, distribution or in any other different area without changing the methodology.

It enhances security and efficiency when compared to the same service executed by aircraft and trained personnel. The final thermal models proved to be useful for both quantitative and qualitative analysis and further fault detection.

Since the thermal inspection is sensitive to infrared reflection from outer sources, it was developed and tested in real scenarios an algorithm to find and eliminate those situations. The results were corroborated by expert measurements with the advantage of the autonomous approach being much faster than traditional ones.

A few extensions are foreseen in this research work. First, the solution will be tested in a wide range of complex scenarios to explore detection of hidden hot spots through the thermal signature of the entire n-dimension temperature vector. Second, it is intended to miniaturize and apply the proposed methodology for aerial inspections in order to inspect areas of difficult access and imminent risk to humans.

## Figures and Tables

**Table 1:** Processes description, priorities and time requirements.

N${}^{\circ }$	Process	Priority	Time Req. (s)	Description
1	Readings	0	0.1	Started by a real time trigger and provides synchronized data acquisition among the sensors
2	Kalman	0	0.01	fuses and updates the position
3	Visual Odometry	1	0.5	uses the corrected position provided by the kalman filter along with visual odometry to improve heading and position
4	Thermal 2D fusion	2	1	responsible to fuse the right visual and thermal images.
5	3D Point Cloud	2	1	generates a dense 3D point cloud through a SLAM algorithm
6	3DT Point Cloud	3	3	reprojects the thermal data into the 3D point cloud. This generates a 4D map
7	MDPC	4	20	accumulates the current 3DT PC to the historical data.

## Appendix A

$I^{th}$	:	$I^{th}:\Omega^{th}\subset Z^{2x2}\to \left[0,2^{12}\right];\left(x,y\right)\mapsto I^{th}\left(x,y\right)$
$I^{v}$	:	$I^{v}:\Omega^{v}\subset Z^{2}\to {\left[0,2^{8}\right]}^{3};\left(x,y\right)\mapsto I^{v}\left(x,y\right)$
**R**	:	R∈O(2)
**t**	:	$t\in R^{2x1}$
$W_{nxn}$	:	$W_{nxn}:\Omega^{w}\subset Z^{2x2}\to {\left[0,2^{8}\right]}^{3};\left(x,y\right)\mapsto W^{v}\left(x,y\right)$
$w_{c}^{\prime }$	:	$w_{c}^{\prime }\in {\left[0,2^{8}\right]}^{3}$
τ	:	$\tau \subset Z^{2}\to \left[0,dr\right];\tau :\left(x_{L}^{v},y_{L}^{v},x_{R}^{v},y_{R}^{v},\right)\mapsto d$
${}_{v}T_{k}^{in}$	:	${}_{v}T_{k}^{in}\in R^{4x4}$
dr	:	dr∈Z
